# Integrated single-cell and transcriptome sequencing data reveal the value of IL1RAP in gastric cancer microenvironment and prognosis

**DOI:** 10.3389/fonc.2025.1584619

**Published:** 2025-05-15

**Authors:** Weifeng Yang, Xiaohua Wu, Jian Wang, Wenquan Ou, Xing Huang

**Affiliations:** Gastrointestinal Surgery, Nanping First Hospital Affiliated to Fujian Medical University, Nanping, Fujian, China

**Keywords:** gastric cancer, machine learning, IL1RAP, tumor microenvironment, M2-type macrophages

## Abstract

**Background:**

Investigating the pivotal role of IL1RAP in the tumor microenvironment of gastric cancer.

**Method:**

Download and collate transcriptomic and single-cell data from gastric cancer patients. Three machine learning algorithms identified distinct sets of prognostic genes in gastric cancer patients. The CIBERSORT and ssGSEA algorithms elucidated immune infiltration patterns, while TIDE and TCGA predicted immune-related outcomes. Furthermore, single-cell sequencing data confirmed the interaction of IL1RAP within the tumor microenvironment. Finally, differential expression levels of IL1RAP protein and mRNA were validated.

**Result:**

After machine learning screening and independent dataset validation, high IL1RAP expression was identified as a poor prognostic factor for gastric cancer patients. Immune infiltration analysis indicated that the low IL1RAP expression group was associated with higher infiltration of CD8+ T cells and M1-type macrophages, whereas the high IL1RAP expression group exhibited increased presence of M2-type macrophages. Immunotherapy prediction models suggested a more favorable response to PD-1 treatment in the low IL1RAP expression group. Prognostic models incorporating IL1RAP demonstrated superior predictive performance. Single-cell data analysis revealed that IL1RAP plays a critical role in regulating intercellular communication within the tumor microenvironment. Our findings were further validated by confirming elevated IL1RAP expression levels in gastric cancer tissues.

**Conclusion:**

IL1RAP plays a critical role in the tumor microenvironment of gastric cancer and serves as a robust predictor of immunotherapy efficacy in gastric cancer.

## Background

Gastric cancer is one of the leading causes of cancer-related deaths worldwide (1). According to the latest report from the National Cancer Organization, millions of new gastric cancer cases are diagnosed annually, with approximately 800,000 related deaths each year (2). Of particular concern is the trend of younger onset ages for gastric cancer (3). Unhealthy lifestyles, including excessive alcohol consumption, smoking, irregular sleep patterns, and high intake of pickled foods, have contributed to a significant rise in incidence rates among individuals in their 30s, increasing from 1% to 4% (4). As a result, gastric cancer now ranks as the third leading cause of cancer-related deaths, coming after lung and colorectal cancers (5).

Symptoms of early gastric cancer are often subtle and may be easily overlooked. Patients may experience non-specific gastrointestinal manifestations such as epigastric discomfort, dull pain, occasional belching, acid reflux, and loss of appetite (6). Early-stage gastric cancer is primarily managed through surgical resection, which offers the potential for curative treatment (7). In comparison, the treatment of advanced-stage gastric cancer generally involves a combination of multiple modalities, such as surgical intervention, chemotherapeutic agents, targeted therapies, and immunotherapies, with the aim of prolonging survival and improving quality of life (8).

IL-1RAP, the interleukin 1 receptor accessory protein, is a critical molecule in the IL-1 signaling pathway (9, 10). It modulates immune cell activity and inflammatory responses by binding to the IL-1 receptor, thereby enhancing IL-1 signaling and activating downstream inflammatory cascades. IL-1RAP is essential for immune regulation, cell proliferation, and differentiation. Aberrant expression of IL-1RAP has been implicated in various diseases, including multiple types of cancer (11–14).

The tumor microenvironment comprises tumor cells, mesenchymal cells, and the extracellular matrix (15). Tumor cells, as the central components, actively proliferate and secrete various signalling molecules, thereby dictating the overall ‘ecological’ dynamics of the microenvironment. In this context, immune cells such as T cells and NK cells fulfill a diverse array of functions, primarily centered on the detection and eradication of tumor cells (16). However, within the gastric cancer microenvironment, tumor cells employ multiple mechanisms to suppress immune cell activity, thereby impairing their ability to perform immune surveillance effectively (17). Moreover, some immune cells are co-opted by tumor cells to support tumor progression, thus facilitating the growth and metastasis of tumor cells (18, 19). Investigating the core regulatory mechanisms within the tumor microenvironment can aid in the development of novel therapeutic strategies. In the context of advanced pancreatic cancer, IL1RAP has been recognized as a key mediator of innate immunity. It plays a critical role in modulating the activation of signaling pathways associated with inflammation and cell proliferation1. Beyond its involvement in the IL-1 receptor pathway, IL1RAP also collaborates with FLT3 and c-KIT—both receptor tyrosine kinases—to facilitate signaling processes and enhance proliferative activities2.

However, current research on IL1RAP in gastric cancer remains limited. In this study, we assessed the potential of IL1RAP as a novel therapeutic target in gastric cancer using machine learning algorithms and immunocorrelation analysis of single-cell data.

## Method

### Data collation

Gastric cancer transcriptomic and clinical data were retrieved from the TCGA database (https://portal.gdc.cancer.gov/). The count data underwent quality control and normalization for differential expression analysis using the ‘DESeq2’ package. The datasets GSE54129, GSE63089, and GSE183904 were retrieved from the GEO database (https://www.ncbi.nlm.nih.gov/geo/). Specifically, GSE54129 and GSE63089 serve as external validation sets to evaluate gene expression and prognosis. Meanwhile, GSE183904 includes single-cell sequencing data from 16 gastric cancer cases, which are utilized for single-cell related analyses.

### Machine learning

Three models, including GBM, RF, and XGB, were utilized to identify prognostic targets in gastric cancer. GBM constructs a strong learner by iteratively adding weak learners (typically decision trees), with each new weak learner specifically designed to correct the errors of its predecessors. On the other hand, a random forest is an ensemble method that comprises multiple decision trees, serving as a classification or regression model based on a tree structure where each internal node represents a test on an attribute, branches represent test outcomes, and leaf nodes represent categories or values. XGBoost employs an additive model, meaning it builds the final model incrementally by adding new weak learners that are optimized based on the existing model to address its deficiencies.

### Function enrichment

Up-regulated and down-regulated genes were firstly analysed by pathway and functional enrichment analyses through the Kyoko Encyclopedia of Genes and Genomes (KEGG) and Gene ontology. GSEA enrichment analysis was used to demonstrate differential gene enrichment phenotypes in the Hallmark gene set.

### Immunological infiltration

The CIBERSORT algorithm and single-sample GSEA (ssGSEA) were employed to evaluate the abundance and functionality of immune cell infiltration for each sample based on the patients’ gene expression profiles. Spearman correlation analyses were conducted to examine the differences in immune cell infiltration levels and functional scores between groups. Additionally, an estimation algorithm was utilized to compare immune scores, stromal scores, and overall scores between the two groups.

### Immunotherapy forecast

TIDE and TCIA are utilized to evaluate the efficacy of immunotherapy. Lower TIDE scores suggest a reduced likelihood of immune escape, thereby indicating higher immunotherapeutic efficacy in patients (http://tide.dfci.harvard.edu). TCIA assesses the potential therapeutic efficacy of immunotherapeutic targets such as PD-1 and CTLA-4 in novel models and further analyzes the potential of immune checkpoint inhibitor genes in therapy. Both platforms ultimately evaluate the functional changes in immune cells that occur during tumor progression.

### Single-cell sequencing data processing

Single-cell sequencing data from 16 gastric cancer samples in GSE183904 were analyzed using R software and relevant packages. The data underwent initial quality control and integration. Quality control criteria included (1): mitochondrial gene content less than 10% (2), total gene expression per cell greater than 300 (3), erythrocyte gene expression less than 8%, and (4) exclusion of cell cycle effects on the results. The Uniform Manifold Approximation and Projection (UMAP) dimensionality reduction technique was employed to visualize the clustered cells on a two-dimensional map. Cell subpopulations were annotated using the ‘SingleR’ package and previous literature.

### Clinical samples

Tumor and surrounding tissue samples were obtained from patients who underwent radical surgery for gastric cancer. Immunohistochemistry and quantitative PCR assays were subsequently performed to verify the differences in IL1RAP expression between tumor and paracancerous tissues. Mutant gastric cancer and paired wild-type gastric cancer paraffin-embedded tumor tissues from 10 patients were obtained. Primary antibodies used for IHC included IL1RAP (Sanying, 30966-1-AP, diluted at 1:1000 for IHC). The procedure was carried out as previously reported. For quantification of IHC images, the IHC Toolbox plugin in ImageJ software (NIH) was utilized. The study was approved by the Ethics Committee of the Nanping First Hospital Affiliated to Fujian Medical University. Informed consent was obtained from the patients for this study. This study was conducted in accordance with good clinical practice and the Declaration of Helsinki, Finland.

### Statistical analysis

To compare the differences between the two groups, the Wilcoxon rank sum test was applied. For prognostic analyses, the Kaplan-Meier method along with the log-rank test was used. All statistical analyses were performed using R software (version 4.1.2, available at https://www.r-project.org/). A two-sided P-value less than 0.05 was considered statistically significant.

## Results

### IL1RAP as a prognostic marker in gastric cancer patients

**Figure 1:**
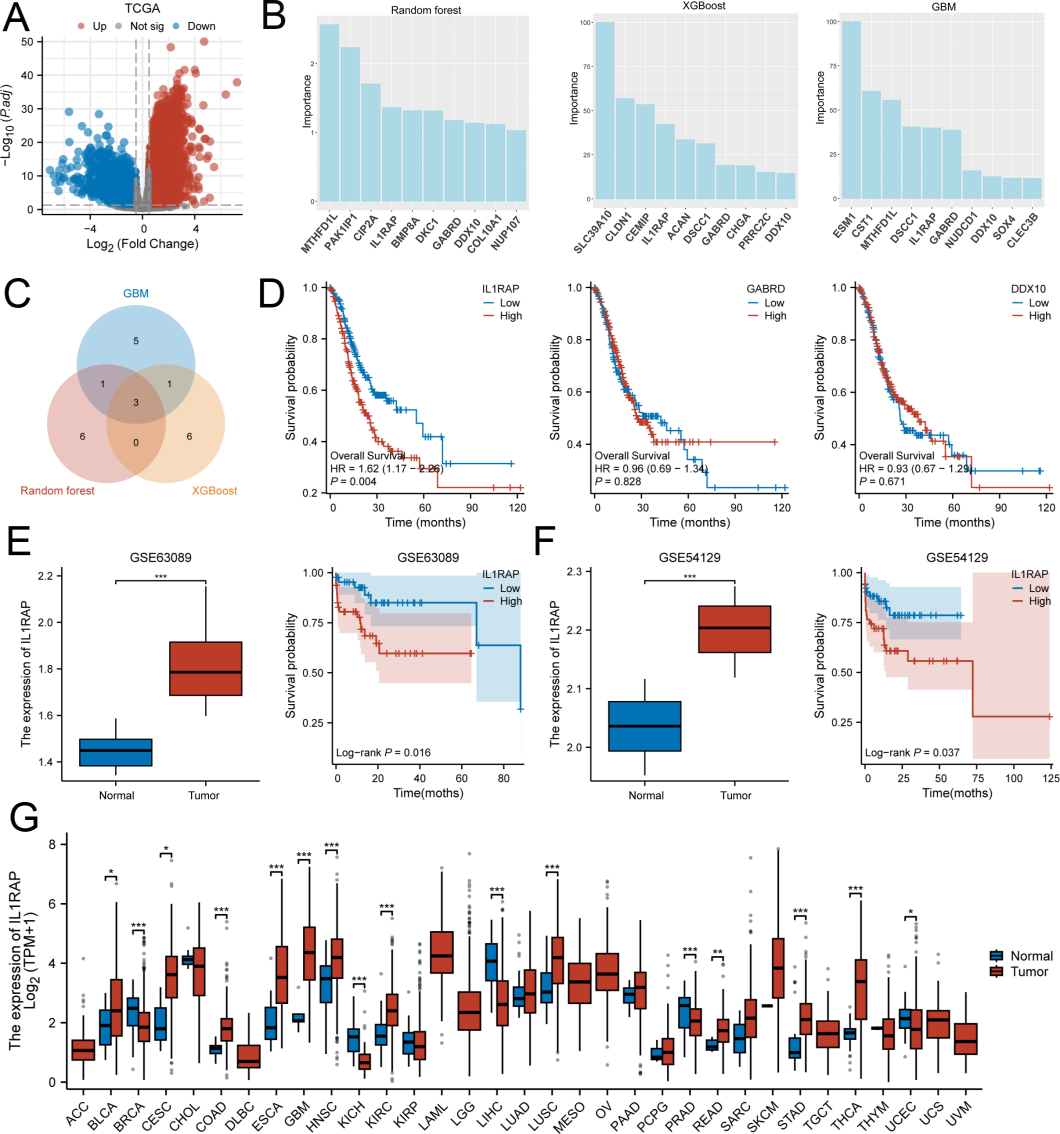
Three machine learning algorithms identified IL1RAP as a characteristic prognostic biomarker for gastric cancer patients. **(A)** Differential expression analysis between TCGA gastric cancer tissues and adjacent non-tumor tissues. **(B)** Utilization of XGBoost, GBM, and Random Forest algorithms to identify prognostic genes for gastric cancer. **(C)** Overlapping results from the three machine learning algorithms. **(D)** Prognostic significance of IL1RAP, CABRD, and DDX10 in gastric cancer patients. **(E, F)** Validation of IL1RAP expression and prognosis using independent datasets GSE63089 and GSE54129. **(G)** Analysis of IL1RAP expression across multiple tumor types. (*P < 0.05; **P < 0.01; ***P < 0.001).

After rigorous screening and quality control, transcriptome data from 36 paraneoplastic tissue samples and 410 gastric cancer tissue samples from TCGA were included in this study. A total of 3,782 differentially expressed genes (**Figure 1A**), detected via differential expression analysis, were utilized in three machine learning algorithms: Gradient Boosting Machine (GBM), Random Forest, and XGBoost. The top ten ranked genes selected by each model were considered for further investigation. After intersecting the results, three genes—IL1RAP, GABRD, and DDX10—were ultimately chosen for subsequent analysis (**Figures 1B, C**). However, survival analysis revealed that GABRD and DDX10 did not exhibit significant differences in gastric cancer prognosis (**Figure 1D**). In contrast, patients with high expression levels of IL1RAP showed significantly worse prognoses compared to those with low expression levels. Validation using independent datasets GSE63089 and GSE54129 confirmed that IL1RAP was not only expressed in paraneoplastic regions but also demonstrated a dose-response relationship between higher expression levels and poorer prognosis (**Figures 1E–G**).

### IL1RAP is implicated in inflammation-related pathways

**Figure 2:**
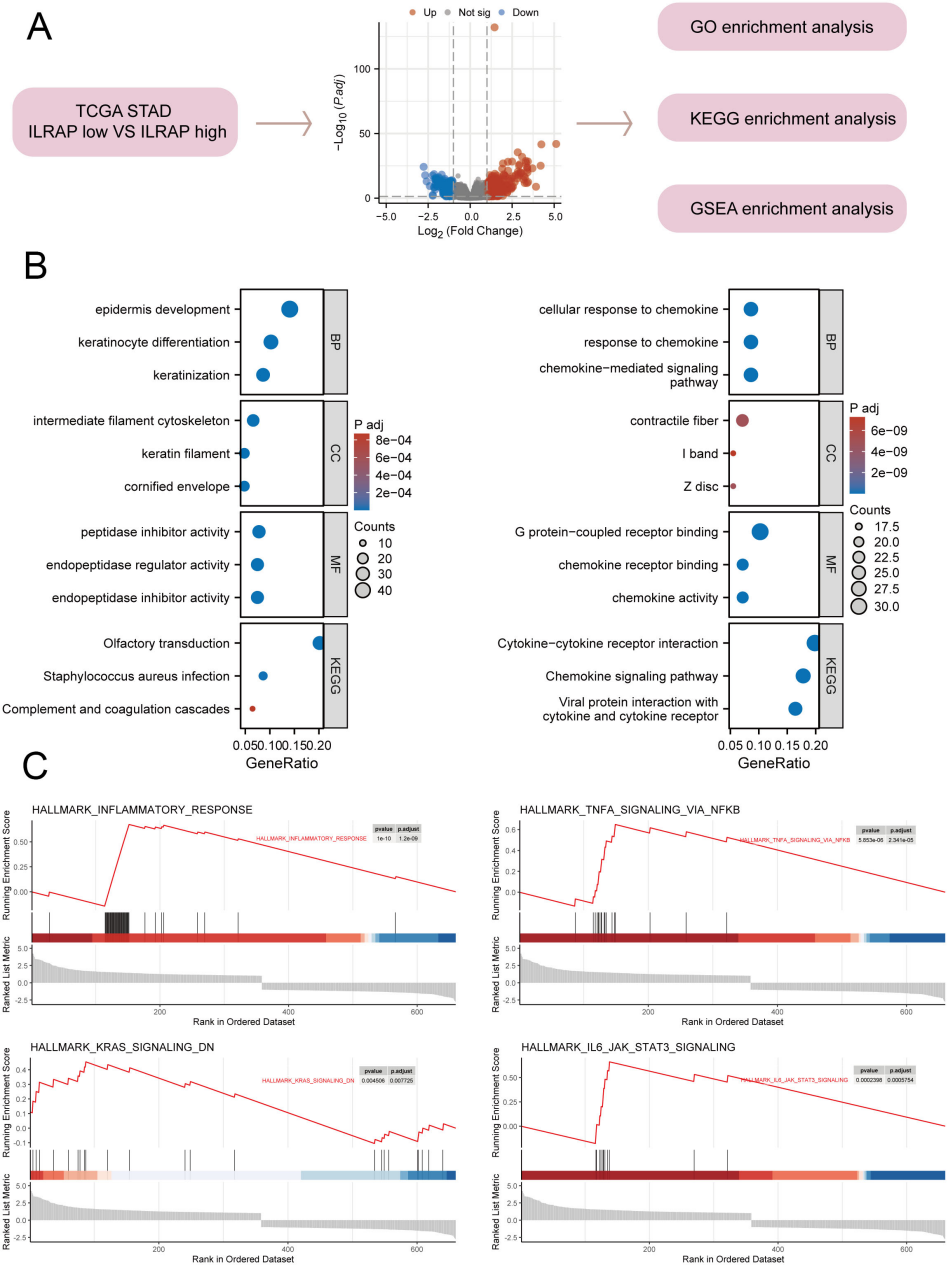
Functional enrichment analysis of IL1RAP in gastric cancer tissues. **(A)** Functional enrichment analysis of genes differentially expressed based on the median difference in IL1RAP expression. **(B)** Functional enrichment analysis of GO and KEGG terms comparing high versus low IL1RAP expression groups. **(C)** GSEA enrichment analysis of IL1RAP-associated differential genes, focusing on immune signaling pathways, including the TNFA signaling pathway, KRAS signaling pathway, and IL6 signaling pathway.

A collection of 410 gastric cancer tissue samples was divided into two groups—those with high IL1RAP expression and those with low IL1RAP expression. The study then examined the distinctions between these two categories. Functional enrichment analysis indicated that IL1RAP was predominantly enriched in pathways related to cytokines, inflammation, and complement activation (**Figures 2A, B**). Additionally, we performed GSEA enrichment analysis using tumor-related phenotypes associated with IL1RAP, which revealed significant enrichment in hallmark pathways such as inflammatory response, TNFA signaling via NFKB, KRAS signaling down, and IL6 JAK-STAT3 signaling via (**Figure 2C**).

### Results of immune infiltration analysis

**Figure 3:**
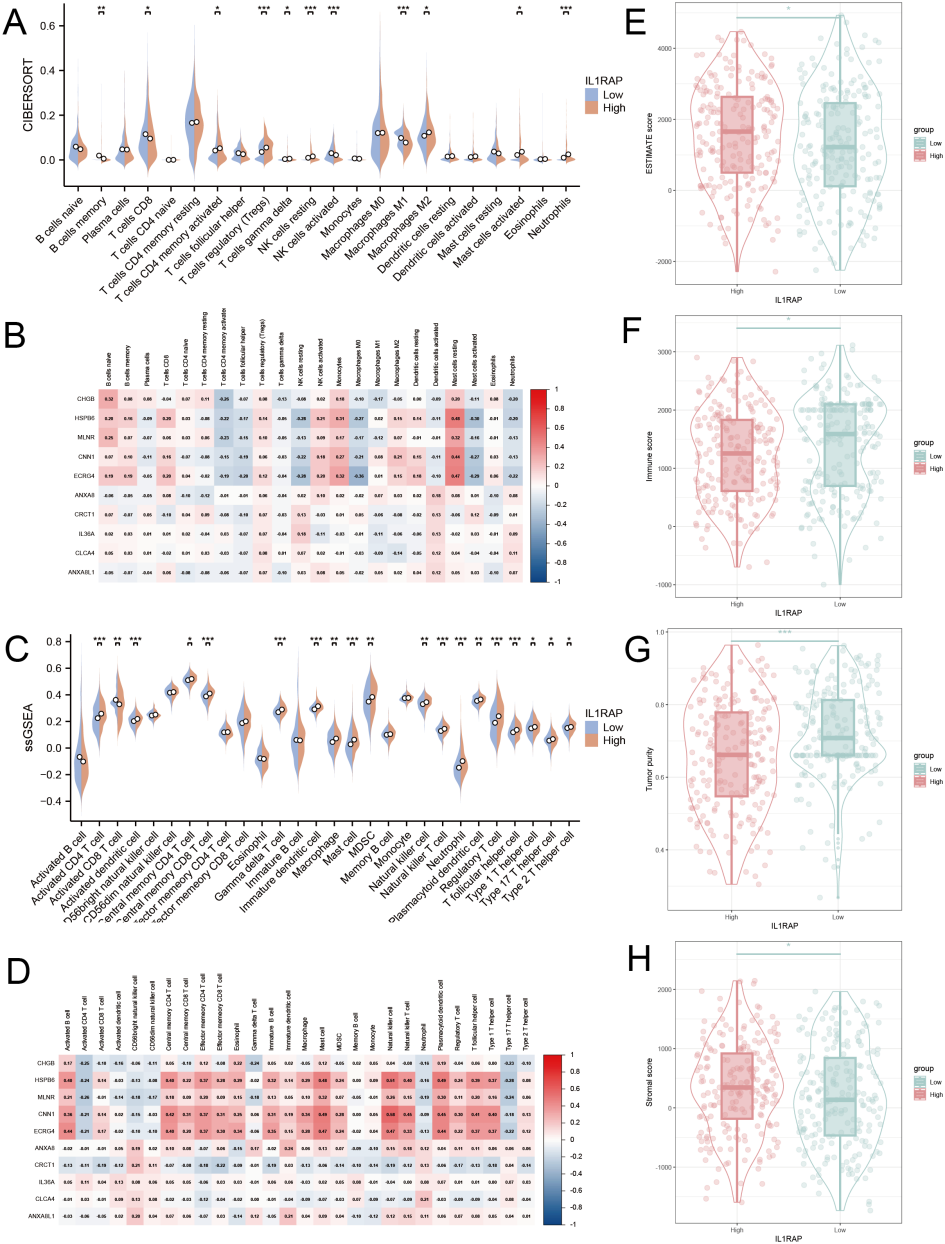
Immunoinfiltration analysis. **(A, B)** The CIBERSORT algorithm elucidates the immune infiltration profiles and correlation analysis with top-ranked genes for both the high and low IL1RAP expression groups. Panels C and D present the ssGSEA algorithm’s findings on immune infiltration and its correlation with leading genes in these expression groups. Panels E, F, G, and H showcase the ESTIMATE algorithm’s assessment of differences in tumor purity score, stromal score, and immune score between the high and low IL1RAP expression groups. (*P < 0.05; **P < 0.01; ***P < 0.001).

Multiple immune infiltration assays were conducted to investigate the association between IL1RAP and immune cells in gastric cancer. The Cibersort algorithm revealed that CD8+ T cells and M1-type macrophages were more abundant in the IL1RAP low-expression group, whereas Tregs and M2-type macrophages were more prevalent in the IL1RAP high-expression group (**Figures 3A, B**). The ssGSEA algorithm indicated that activated CD8+ T cells exhibited greater functionality in the IL1RAP low-expression group, while MDSCs showed enhanced functionality in the IL1RAP high-expression group (**Figures 3C, D**). A strong correlation was observed between the top-ranked differentially expressed genes and the abundance and function of infiltrating immune cells. Additionally, the ESTIMATE algorithm demonstrated that IL1RAP expression was associated with higher immune scores and intermediate stromal scores in the high-expression group (**Figures 3E–H**).

### Immunotherapy predictions

**Figure 4:**
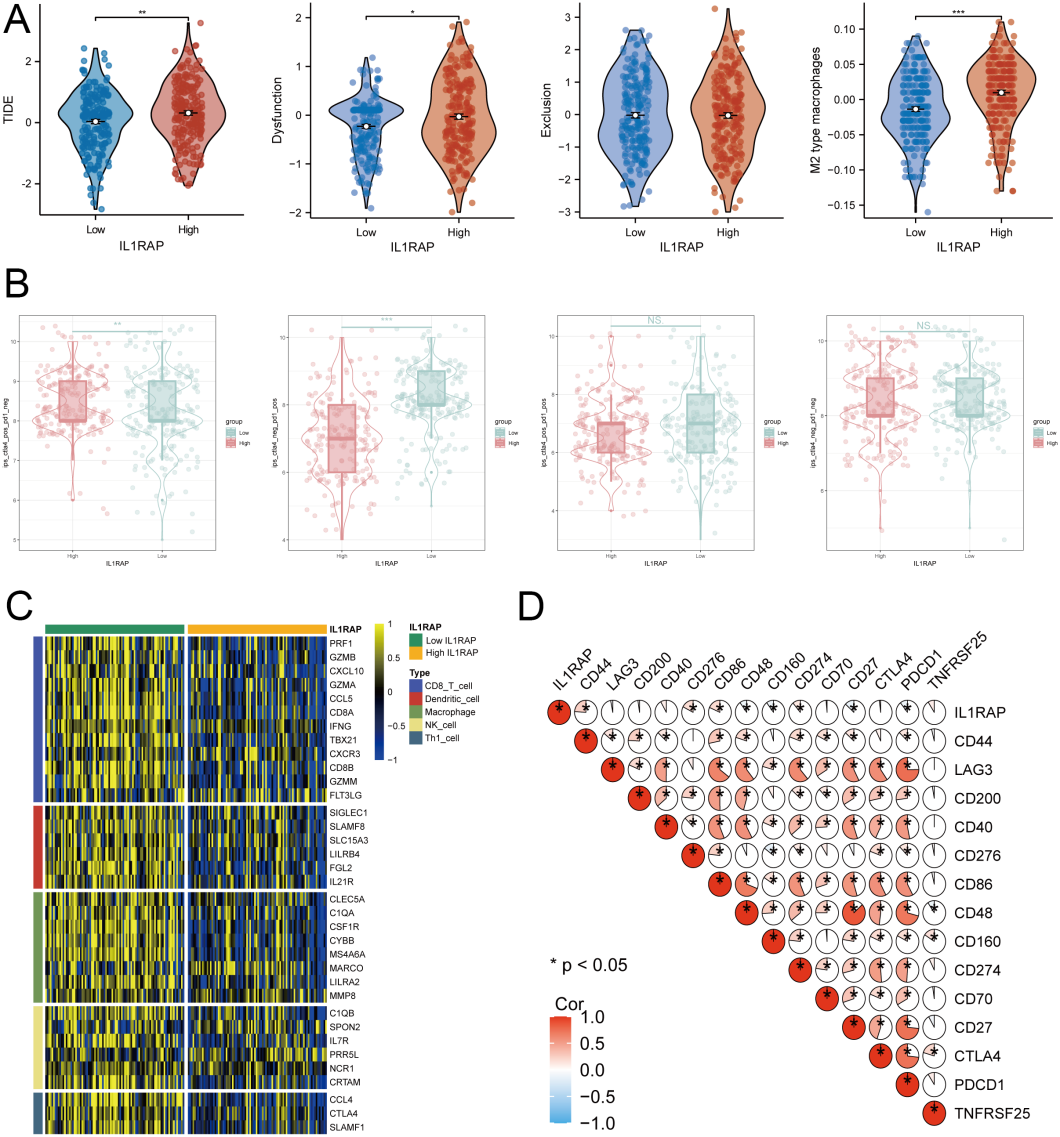
Immunotherapy Outcome Prediction. **(A)** The TIDE method is utilized to forecast the outcomes of immunotherapy for patients. **(B)** The TCIA algorithm predicts treatment outcomes for patients receiving PD-1 and CTLA-4 immunotherapies. **(C)** Significant differences in the changes of key immune cell function genes are observed between IL1RAP high-expression and low-expression groups. **(D)** Correlation analysis between IL1RAP expression and immune checkpoint inhibitor gene expression. (*P < 0.05; **P < 0.01; ***P < 0.001).

In patients with high IL1RAP expression, TIDE scores were significantly elevated, suggesting less favorable outcomes for immunotherapy. The high-expression group also exhibited increased CTL dysfunction and higher M2 macrophage scores compared to the low-expression group (**Figure 4A**). According to the TIDE immunotherapy prediction model, the CTLA4 treatment scores were notably higher in the high IL1RAP expression group, while the PD-1 treatment scores were more favorable in the low IL1RAP expression group. Moreover, the functionality of critical immune cells, such as CD8^+^ T cells and dendritic cells, was reduced in the high IL1RAP expression group relative to the low-expression group. Additionally, a significant correlation was observed between IL1RAP expression levels and various immune checkpoint inhibitory genes (**Figures 4B–D**).

### Clinical prognostic models

**Figure 5:**
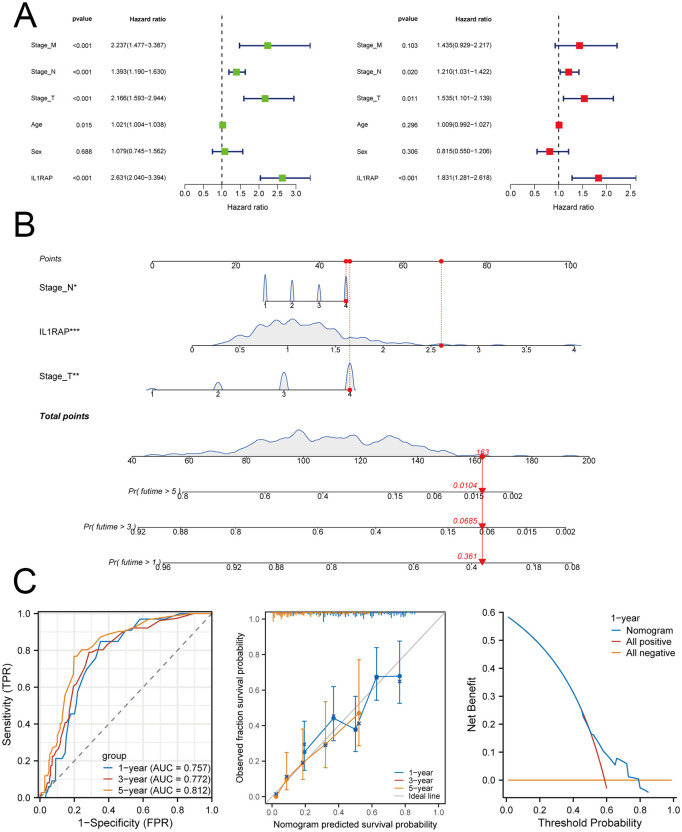
IL1RAP in conjunction with clinical variables for prognostic modeling. **(A)** Results of univariate and multivariate Cox regression analyses. **(B)** A prognostic nomogram was developed based on the results of the multivariate Cox regression analysis. **(C)** Time-dependent ROC curves, calibration plots, and decision curve analysis (DCA) were utilized to evaluate the performance of the model.

Univariate and multivariate Cox regression analyses revealed that IL1RAP serves as an independent prognostic marker for gastric cancer. We constructed a prognostic nomogram designed for patients with gastric cancer. The model’s predictive accuracy was validated through time-dependent ROC curves, calibration plots, and decision curve analysis, all of which indicated strong and reliable performance (**Figure 5**).

### Single-cell data analysis

**Figure 6:**
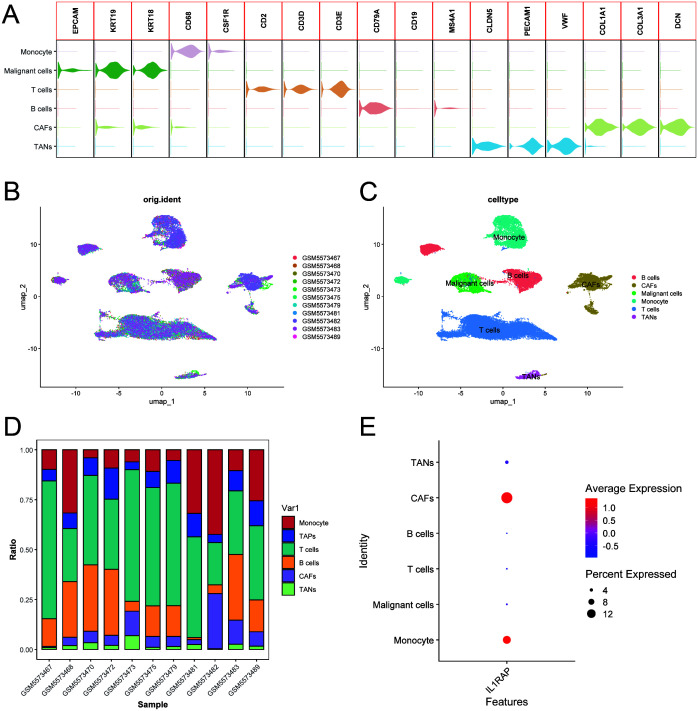
Integration of single-cell data from gastric cancer elucidates the role of IL1RAP. **(A)** Summary of marker genes for each cell type according to previous studies. **(B, C)** Integrated clustering analysis of single-cell data from 11 cases of gastric cancer. **(D)** Heatmap representation of cell type distribution in 11 cases of gastric cancer. **(E)** IL1RAP expression was predominantly observed in cancer-associated fibroblasts (CAFs) and other specific cell types.

**Figure 7:**
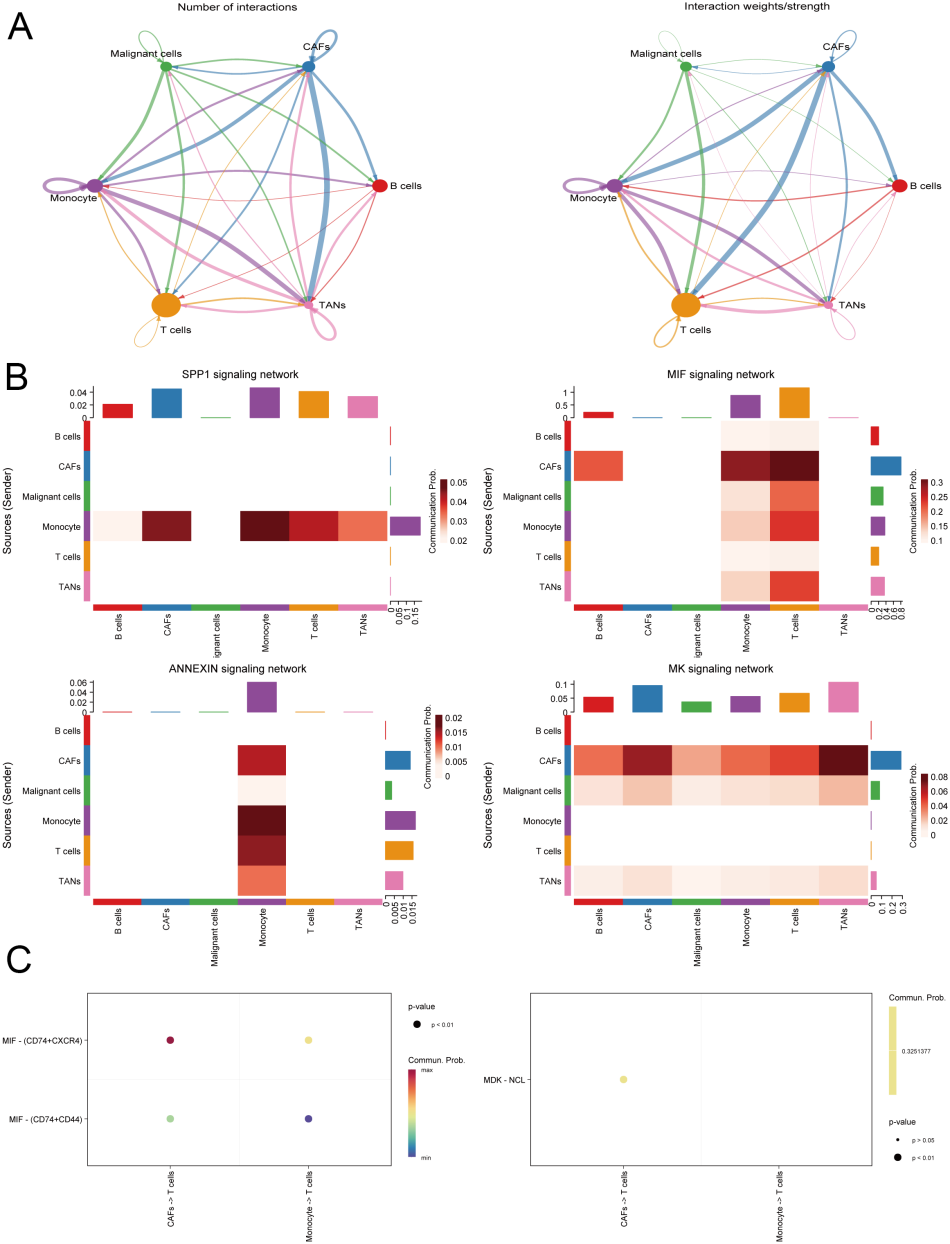
Analysis of intercellular communication within the gastric cancer microenvironment. **(A)** Evaluation of the strength and density of intercellular communication in the gastric cancer tumor microenvironment. **(B)** Intercellular communication predominantly involves inflammation-related pathways. **(C)** Identification and demonstration of receptors and ligands involved in intercellular communication.

We integrated single-cell sequencing data from 11 gastric cancer patients. Based on the expression profiles of marker genes, we identified six distinct cell types: B cells, T cells, monocytes, cancer-associated fibroblasts (CAFs), malignant cells, and tumor-associated neutrophils (TANs) (**Figure 6**). The tumor microenvironments of these samples exhibited heterogeneity in cellular composition. IL1RAP was predominantly expressed in cancer-associated fibroblasts (CAFs) and played a critical role in regulating solid tumors. Analysis of intercellular communication revealed significant activity in multiple signaling pathways between mesenchymal and immune cells, including the SPP1, MIF, MAPK, and TNF signaling pathways (**Figure 7**).

### Expression validation

**Figure 8:**
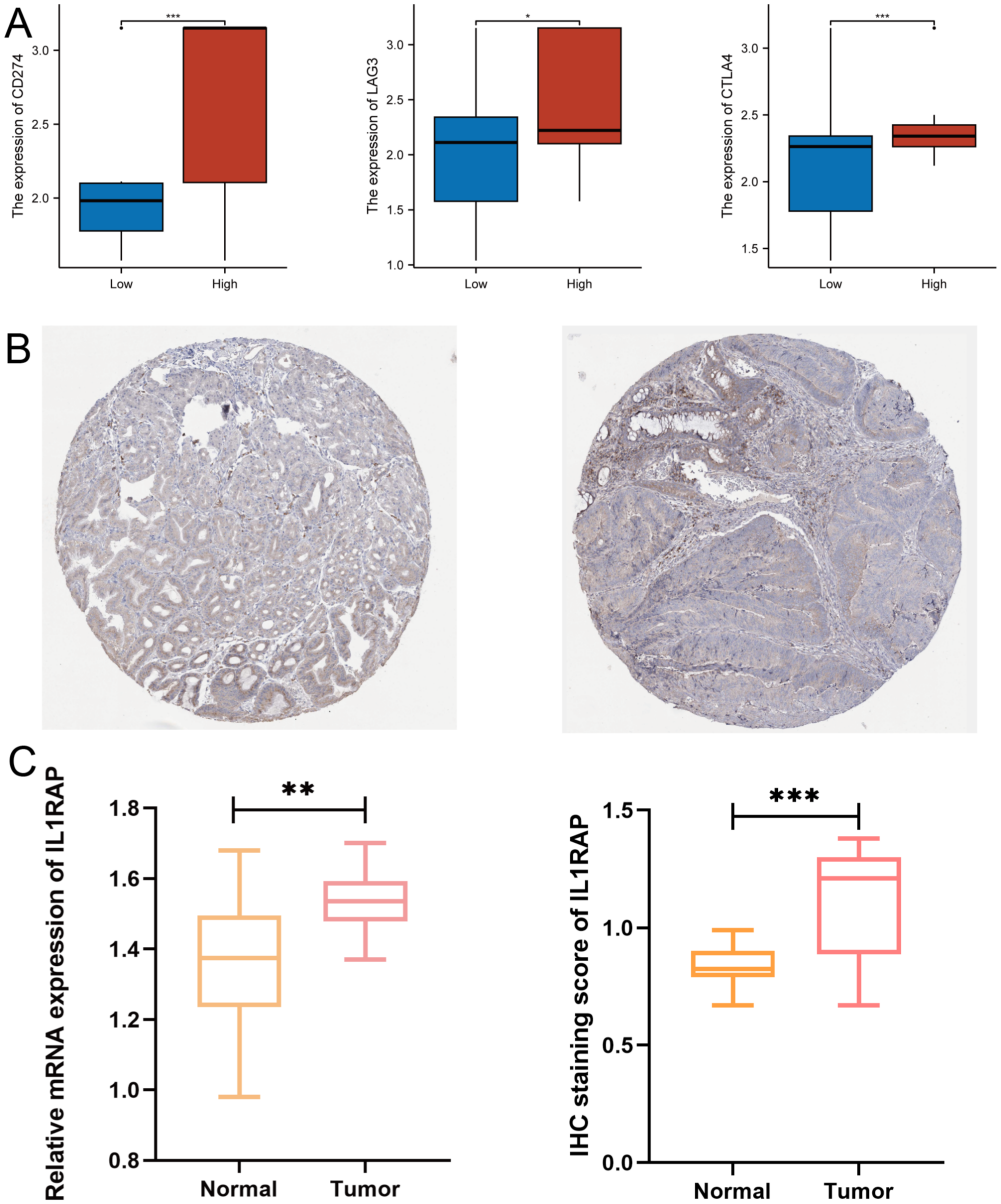
Validation of Protein and mRNA Expression. **(A)** Differential expression of immune checkpoint genes between the IL1RAP high-expression and low-expression groups. **(B)** Immunohistochemical analysis of normal versus paracancerous tissues. **(C)** Comparative analysis of IL1RAP protein and mRNA expression levels. (*P < 0.05; **P < 0.01; ***P < 0.001).

We validated the expression of IL1RAP at both the mRNA and protein levels. The results demonstrated that both the mRNA and protein levels of IL1RAP were significantly upregulated in gastric cancer tissues. These findings were consistent with data from public databases (**Figure 8**).

## Discussion

The occurrence and development of gastric cancer is a multifaceted process influenced by genetic, environmental, and lifestyle factors, as well as Helicobacter pylori infection (20, 21). Recent advancements in molecular biology and genomics have deepened our understanding of the molecular characteristics of gastric cancer. Molecular typing studies have provided novel insights for personalized treatment strategies (22). For instance, The Cancer Genome Atlas (TCGA) project classified gastric cancer into four unique molecular categories: those positive for Epstein-Barr Virus (EBV), microsatellite instability-high (MSI-H), genomically stable, and chromosomally unstable (23). These subtypes exhibit differences not only in biological behavior but also in their responsiveness to chemotherapy, targeted therapy, and immunotherapy (24). Despite significant progress in gastric cancer research, numerous challenges persist. Key issues include improving early diagnosis rates, overcoming tumor heterogeneity and treatment resistance, and optimizing immunotherapy strategies (25, 26). Further investigation into the key drivers of gastric cancer development is crucial for developing more precise therapeutic approaches (27).

IL-1RAP plays a crucial role in mediating the IL-1 signaling pathway, which is implicated in inflammation, cancer, neurological disorders, metabolic diseases, and infectious diseases (28). Our study revealed that IL1RAP expression is significantly elevated in gastric cancer at both protein and mRNA levels, which correlates with a poorer prognosis in patients. We conducted an in-depth analysis of the role of IL1RAP within the gastric cancer microenvironment. Consistent with our findings, numerous studies have likewise demonstrated that IL1RAP and immune cells are closely interconnected. Rehman demonstrated that patients in the IL1RAP high-expression group exhibited a worse prognosis and a positive correlation with tumor stem cell activity, findings that were validated in two independent knockdown cell lines (29). Zhou et al. further revealed that IL1RAP mediates the interaction between tumor cells and M2-type macrophages following PD-1 treatment, which subsequently alters the neutrophil-to-lymphocyte ratio, contributing to an unfavorable prognosis for patients (30). In chronic inflammatory conditions, IL-1RAP binds to the IL-1 receptor, thereby activating the NF-κB and MAPK signaling pathways. This activation promotes the release of pro-inflammatory cytokines such as IL-6 and TNF-α, ultimately resulting in synovial inflammation and cartilage destruction (13, 31). Additionally, IL-1RAP enhances IL-1β signaling in intestinal epithelial cells and immune cells, including macrophages and T cells, thereby exacerbating inflammatory responses (12, 32). This leads to impaired intestinal barrier function and sustained chronic inflammation (33). Our results reveal a complex relationship between M1 and M2 macrophages and IL1RAP expression. Notably, we observed a greater infiltration of M1 macrophages in the group with lower IL1RAP expression than in the group with higher IL1RAP expression. Conversely, M2 macrophages exhibited a preference for the high IL1RAP expression group. These observations were corroborated by single-cell data analysis, which validated the results obtained from transcriptome data.

IL1RAP is highly expressed in various cancers and plays a multifaceted role in promoting tumor growth, metastasis, and immune escape. IL-1RAP plays a multifaceted and complex role in tumor progression and regulation. In leukaemia, IL1RAP is prominently expressed on the surface of leukaemia stem cells, where it drives cell proliferation and survival via activation of the IL-1 signalling pathway (34). Additionally, IL1RAP facilitates immune evasion by modulating the inflammatory response within the tumor microenvironment (35). The role of IL1RAP in solid malignancies is even more intricate. In glioblastoma, IL1RAP, which is highly expressed in glioblastoma stem cells, sustains self-renewal and tumourigenic potential through the activation of Wnt/β-catenin and Notch signalling pathways (36). Furthermore, IL1RAP enhances inflammatory responses in the tumor microenvironment by mediating the pro-inflammatory effects of IL-1β, thereby supporting tumor growth and invasion (37). Nuclear factor-κB plays a crucial role in the tumor microenvironment. Liu et al. demonstrated that IL1RAP upregulates nuclear factor-κB signaling, thereby promoting CD47 expression and subsequently inhibiting macrophage phagocytosis. This indicates that nuclear factor-κB may serve as a key pathway through which IL1RAP mediates functional alterations in both M1 and M2 macrophages, a conclusion that aligns with our findings (38). In pancreatic cancer, IL1RAP promotes the activation of tumor-associated fibroblasts and immunosuppressive cells (e.g., MDSCs, Tregs), fostering an immunosuppressive microenvironment through the activation of the IL-1 signalling pathway (39). Moreover, IL1RAP enhances the migration and invasion of pancreatic cancer cells by inducing epithelial-mesenchymal transition (40). It has also been shown that IL1RAP contributes to chemoresistance in pancreatic cancer cells, such as resistance to gemcitabine, by activating NF-κB and STAT3 signalling pathways (41).

However, this study still has several limitations. Firstly, the effect of IL1RAP on macrophage differentiation in gastric cancer has not been validated at the animal level. Secondly, further exploration is needed to fully understand the mechanisms by which IL1RAP is involved in gastric cancer. Finally, the therapeutic efficacy of IL1RAP knockdown on gastric cancer should be confirmed through animal studies.

## Concluson

IL1RAP plays a critical role in the tumor microenvironment of gastric cancer and serves as a robust predictor of immunotherapy efficacy in patients. Therefore, IL1RAP is anticipated to be a promising target for enhancing gastric cancer immunotherapy.

## Data Availability

The original contributions presented in the study are included in the article/supplementary material. Further inquiries can be directed to the corresponding author.
