# Supplementary material for: Integrated single-cell and transcriptome sequencing data reveal the value of IL1RAP in gastric cancer microenvironment and prognosis
Source: Front Oncol. 2025 May 15;15:1584619. doi: 10.3389/fonc.2025.1584619 (PMC12119286; doi:10.3389/fonc.2025.1584619)
Supplement: Supplementary file 1 [file Table1.docx]

library(readr)

#install.packages("parallel")

library(VIM)

library(caret)

library(rpart)

library(rpart.plot)

library(Metrics)

library(stringr)

library(rpart)

library(tibble)

library(bitops)

library(rattle)

library(rpart.plot)

library(RColorBrewer)

library(tidyverse)

library(limma)

library(pheatmap)

library(visNetwork)

library(ggpol)

library(ggplot2)

library(sparkline)

library(dplyr)

library(tidyverse)

library(caret)

library(DALEX)

library(gbm)

library(caret)

library(parallel)

# 定义一个简单的函数

myFunction <- function(x) {

return(x^2)

}

# 获取 CPU 核心数

numCores <- detectCores()

# 创建一个集群

cl <- makeCluster(numCores)

# 使用 parLapply 函数并行执行 myFunction 函数

results <- parLapply(cl, 1:20, myFunction)

# 停止集群

stopCluster(cl)

print(results)

setwd("D:\\Download\\1.diannaozidai\\1.zhuomian\\杂七杂八\\刘师兄\\2.胃癌\\机器学习\\1.GBM")

#####数据准备，行名是样本名，列名是基因名

data<-read.csv(file="diff.csv", row.names=1,check.names=F)

data<-data.frame(t(data))

group=gsub("(.*)\\-(.*)", "\\2", row.names(data))

set.seed(1234)

metric <- "RMSE"

myControl <- trainControl(method="cv", number=5)

# Fitting model

system.time(fitControl <- trainControl( method = "repeatedcv", number = 4, repeats = 4))

system.time(fit <- train(x=data,y=as.factor(group), method = "gbm", trControl = fitControl,verbose = FALSE))

#绘制基因重要性梯度图

importances <- varImp(fit)

importances

importance <- as.data.frame(importances$importance)

importance$gene<-row.names(importance)

importance<-importance[order(-importance$Overall), ]

write.csv(importance,"GBM.csv",quote = FALSE)

write.table(importance,"GBM2.txt")

#输入完上面那串代码后，显示的结果就是GBM结果，将他们复制出来。

#重要性筛选区域设置多少可以自己定，我这里是只要重要性不为0都可以

#删除为重要性为0的gene后重新导入

a<-importance

a<-read.csv("GBM.csv",row.names = 1)

varimpdf <- data.frame(var = row.names(a),

impor = a[,1])

ggplot(varimpdf,aes(x = reorder(var,-impor), y = impor))+

geom_col(colour = "lightblue",fill = "lightblue")+

labs(title="Feature gene importance (Gradient Boosting Machine)", x="",y = "importance")+

theme(plot.title = element_text(size=12,hjust=0.5))+

theme(axis.text.x = element_text(size = 5))+

theme(axis.text.y = element_text(size = 12))+

theme(axis.text.x = element_text(angle = 50,vjust = 0.85,hjust = 0.75))

#引用包

install.packages("randomForest")

library(randomForest)

set.seed(123456)

inputFile="diffGeneExp.txt" #输入文件

setwd("D:\\Download\\1.diannaozidai\\1.zhuomian\\杂七杂八\\刘师兄\\2.胃癌\\机器学习\\2.RF")

#读取输入文件

data<-read.csv(file="diff.csv", row.names=1,check.names=F)

data<-data.frame(t(data))

group=gsub("(.*)\\-(.*)", "\\2", row.names(data))

#随机森林树

rf=randomForest(as.factor(group)~., data=data, ntree=100)

pdf(file="森林.pdf", width=6, height=6)

plot(rf, main="Random forest", lwd=2)

dev.off()

#找出误差最小的点

optionTrees=which.min(rf$err.rate[,1])

optionTrees

rf2=randomForest(as.factor(group)~., data=data, ntree=optionTrees)

#查看基因的重要性

importance=importance(x=rf2)

#绘制基因的重要性图

pdf(file="GeneIm.pdf", width=6, height=8)

varImpPlot(rf2, main="")

dev.off()

#挑选疾病特征基因

rfGenes=importance[order(importance[,"MeanDecreaseGini"], decreasing = TRUE),]

rfGenes=names(rfGenes[rfGenes>0]) #挑选重要性评分大于2的基因

rfGenes=names(rfGenes[1:100]) #挑选重要性评分最高的30个基因

rfGenes<-data.frame(rfGenes)

write.csv(rfGenes, file="随机森林Genes.csv")

write.csv(importance, file="随机森林Genes.csv")

rfGenes<-data.frame(rfGenes)

rfGenes$gene<-row.names(rfGenes)

rfGenes<-rfGenes[order(-rfGenes$rfGenes), ] # -是降序，不加是正序

write.csv(rfGenes,"rf.csv",quote = FALSE)

write.table(importance,"GBM2.txt")

#输出重要基因的表达量

sigExp=t(data[,rfGenes])

sigExpOut=rbind(ID=colnames(sigExp),sigExp)

write.table(sigExpOut, file="imGeneExp.txt", sep="\t", quote=F, col.names=F)

install.packages("xgboost")

library(xgboost)

library(caret)

library(tidyverse)

library(readr)

library(VIM)

library(caret)

library(rpart)

library(rpart.plot)

library(Metrics)

library(stringr)

library(rpart)

library(tibble)

library(bitops)

library(rattle)

library(rpart.plot)

library(RColorBrewer)

library(tidyverse)

library(limma)

library(pheatmap)

library(visNetwork)

library(ggpol)

library(ggplot2)

library(sparkline)

library(dplyr)

library(tidyverse)

library(caret)

library(DALEX)

library(gbm)

set.seed(123)

setwd("D:\\Download\\1.diannaozidai\\1.zhuomian\\杂七杂八\\刘师兄\\2.胃癌\\机器学习\\3.XGB")

data<-read.csv(file="diff.csv", row.names=1,check.names=F)

data<-data.frame(t(data))

group=gsub("(.*)\\-(.*)", "\\2", row.names(data))

# Fitting model(用caret实现)

TrainControl <- trainControl( method = "repeatedcv", number = 10, repeats = 4)

model<- train(x=data,y=as.factor(group), method = "xgbTree", trControl = TrainControl,verbose = FALSE)

plot(varImp(model))

importance <- varImp(model)

head(importance)

important <- as.data.frame(importance$importance)

a<-important

varimpdf <- data.frame(var = row.names(a),

impor = a[,1])

varimpdf1<- varimpdf[varimpdf$impor>5,]

write.csv(varimpdf,"XGB.csv")

ggplot(varimpdf1,aes(x = reorder(var,-impor), y = impor))+

geom_col(colour = "lightblue",fill = "lightblue")+

labs(title="Feature gene importance (XGBoost)", x="",y = "importance")+

theme(plot.title = element_text(size=12,hjust=0.5))+

theme(axis.text.x = element_text(size = 3))+

theme(axis.text.y = element_text(size = 12))+

theme(axis.text.x = element_text(angle = 50,vjust = 0.85,hjust = 0.75))

################单细胞################

##############################第二种方法harmony###############

install.packages("devtools")

library(devtools)

install_github("immunogenomics/harmony")

library(harmony)

BiocManager::install("SingleCellExperiment")

install.packages('Seurat')

getwd()

BiocManager::install("SingleCellExperiment")

setwd("D:\\Download\\1.diannaozidai\\1.zhuomian\\杂七杂八\\刘师兄\\2.胃癌\\Single-cell\\2.分析")

###加载所需要的包

library(Seurat)

library(tidyverse)

library(dplyr)

library(patchwork)

#install.packages("devtools")

library(devtools)

#install_github("immunogenomics/harmony")

library(harmony)

scRNA<- read.csv("GSM5573489_sample24.csv.gz",stringsAsFactors = FALSE, row.names = 1)

scRNA.1<- CreateSeuratObject(counts = scRNA, project = "GSM5573489", min.cells = 3, min.features = 200)

scRNA<- read.csv("GSM5573467_sample2.csv.gz",stringsAsFactors = FALSE, row.names = 1)

scRNA.2<- CreateSeuratObject(counts = scRNA, project = "GSM5573467", min.cells = 3, min.features = 200)

scRNA<- read.csv("GSM5573468_sample3.csv.gz",stringsAsFactors = FALSE, row.names = 1)

scRNA.3<- CreateSeuratObject(counts = scRNA, project = "GSM5573468", min.cells = 3, min.features = 200)

scRNA<- read.csv("GSM5573470_sample5.csv.gz",stringsAsFactors = FALSE, row.names = 1)

scRNA.4<- CreateSeuratObject(counts = scRNA, project = "GSM5573470", min.cells = 3, min.features = 200)

scRNA<- read.csv("GSM5573472_sample7.csv.gz",stringsAsFactors = FALSE, row.names = 1)

scRNA.5<- CreateSeuratObject(counts = scRNA, project = "GSM5573472", min.cells = 3, min.features = 200)

scRNA<- read.csv("GSM5573473_sample8.csv.gz",stringsAsFactors = FALSE, row.names = 1)

scRNA.6<- CreateSeuratObject(counts = scRNA, project = "GSM5573473", min.cells = 3, min.features = 200)

scRNA<- read.csv("GSM5573475_sample10.csv.gz",stringsAsFactors = FALSE, row.names = 1)

scRNA.7<- CreateSeuratObject(counts = scRNA, project = "GSM5573475", min.cells = 3, min.features = 200)

scRNA<- read.csv("GSM5573477_sample12.csv.gz",stringsAsFactors = FALSE, row.names = 1)

scRNA.8<- CreateSeuratObject(counts = scRNA, project = "GSM5573477", min.cells = 3, min.features = 200)

scRNA<- read.csv("GSM5573478_sample13.csv.gz",stringsAsFactors = FALSE, row.names = 1)

scRNA.9<- CreateSeuratObject(counts = scRNA, project = "GSM5573478", min.cells = 3, min.features = 200)

scRNA<- read.csv("GSM5573479_sample14.csv.gz",stringsAsFactors = FALSE, row.names = 1)

scRNA.10<- CreateSeuratObject(counts = scRNA, project = "GSM5573479", min.cells = 3, min.features = 200)

scRNA<- read.csv("GSM5573480_sample15.csv.gz",stringsAsFactors = FALSE, row.names = 1)

scRNA.11<- CreateSeuratObject(counts = scRNA, project = "GSM5573480", min.cells = 3, min.features = 200)

scRNA<- read.csv("GSM5573481_sample16.csv.gz",stringsAsFactors = FALSE, row.names = 1)

scRNA.12<- CreateSeuratObject(counts = scRNA, project = "GSM5573481", min.cells = 3, min.features = 200)

scRNA<- read.csv("GSM5573482_sample17.csv.gz",stringsAsFactors = FALSE, row.names = 1)

scRNA.13<- CreateSeuratObject(counts = scRNA, project = "GSM5573482", min.cells = 3, min.features = 200)

scRNA<- read.csv("GSM5573483_sample18.csv.gz",stringsAsFactors = FALSE, row.names = 1)

scRNA.14<- CreateSeuratObject(counts = scRNA, project = "GSM5573483", min.cells = 3, min.features = 200)

scRNA<- read.csv("GSM5573484_sample19.csv.gz",stringsAsFactors = FALSE, row.names = 1)

scRNA.15<- CreateSeuratObject(counts = scRNA, project = "GSM5573484", min.cells = 3, min.features = 200)

scRNA<- read.csv("GSM5573485_sample20.csv.gz",stringsAsFactors = FALSE, row.names = 1)

scRNA.16<- CreateSeuratObject(counts = scRNA, project = "GSM5573485", min.cells = 3, min.features = 200)

scRNA.1[["percent.mt"]] <- PercentageFeatureSet(scRNA.1, pattern = "^MT-")

scRNA.2[["percent.mt"]] <- PercentageFeatureSet(scRNA.2, pattern = "^MT-")

scRNA.3[["percent.mt"]] <- PercentageFeatureSet(scRNA.3, pattern = "^MT-")

scRNA.4[["percent.mt"]] <- PercentageFeatureSet(scRNA.4, pattern = "^MT-")

scRNA.5[["percent.mt"]] <- PercentageFeatureSet(scRNA.5, pattern = "^MT-")

scRNA.6[["percent.mt"]] <- PercentageFeatureSet(scRNA.6, pattern = "^MT-")

scRNA.7[["percent.mt"]] <- PercentageFeatureSet(scRNA.7, pattern = "^MT-")

scRNA.13[["percent.mt"]] <- PercentageFeatureSet(scRNA.13, pattern = "^MT-")

scRNA.14[["percent.mt"]] <- PercentageFeatureSet(scRNA.14, pattern = "^MT-")

scRNA.10[["percent.mt"]] <- PercentageFeatureSet(scRNA.10, pattern = "^MT-")

scRNA.11[["percent.mt"]] <- PercentageFeatureSet(scRNA.11, pattern = "^MT-")

scRNA.12[["percent.mt"]] <- PercentageFeatureSet(scRNA.12, pattern = "^MT-")

HB.genes <- c("HBA1","HBA2","HBB","HBD","HBE1","HBG1","HBG2","HBM","HBQ1","HBZ")

HB_m <- match(HB.genes, rownames(scRNA.1@assays$RNA))

HB.genes <- rownames(scRNA.1@assays$RNA)[HB_m]

HB.genes <- HB.genes[!is.na(HB.genes)]

scRNA.1[["percent.HB"]]<-PercentageFeatureSet(scRNA.1, features=HB.genes)

col.num <- length(levels(scRNA.1@active.ident))

HB.genes <- c("HBA1","HBA2","HBB","HBD","HBE1","HBG1","HBG2","HBM","HBQ1","HBZ")

HB_m <- match(HB.genes, rownames(scRNA.2@assays$RNA))

HB.genes <- rownames(scRNA.2@assays$RNA)[HB_m]

HB.genes <- HB.genes[!is.na(HB.genes)]

scRNA.2[["percent.HB"]]<-PercentageFeatureSet(scRNA.2, features=HB.genes)

col.num <- length(levels(scRNA.2@active.ident))

HB.genes <- c("HBA1","HBA2","HBB","HBD","HBE1","HBG1","HBG2","HBM","HBQ1","HBZ")

HB_m <- match(HB.genes, rownames(scRNA.3@assays$RNA))

HB.genes <- rownames(scRNA.3@assays$RNA)[HB_m]

HB.genes <- HB.genes[!is.na(HB.genes)]

scRNA.3[["percent.HB"]]<-PercentageFeatureSet(scRNA.3, features=HB.genes)

col.num <- length(levels(scRNA.3@active.ident))

HB.genes <- c("HBA1","HBA2","HBB","HBD","HBE1","HBG1","HBG2","HBM","HBQ1","HBZ")

HB_m <- match(HB.genes, rownames(scRNA.4@assays$RNA))

HB.genes <- rownames(scRNA.4@assays$RNA)[HB_m]

HB.genes <- HB.genes[!is.na(HB.genes)]

scRNA.4[["percent.HB"]]<-PercentageFeatureSet(scRNA.4, features=HB.genes)

col.num <- length(levels(scRNA.4@active.ident))

HB.genes <- c("HBA1","HBA2","HBB","HBD","HBE1","HBG1","HBG2","HBM","HBQ1","HBZ")

HB_m <- match(HB.genes, rownames(scRNA.5@assays$RNA))

HB.genes <- rownames(scRNA.5@assays$RNA)[HB_m]

HB.genes <- HB.genes[!is.na(HB.genes)]

scRNA.5[["percent.HB"]]<-PercentageFeatureSet(scRNA.5, features=HB.genes)

col.num <- length(levels(scRNA.5@active.ident))

HB.genes <- c("HBA1","HBA2","HBB","HBD","HBE1","HBG1","HBG2","HBM","HBQ1","HBZ")

HB_m <- match(HB.genes, rownames(scRNA.6@assays$RNA))

HB.genes <- rownames(scRNA.6@assays$RNA)[HB_m]

HB.genes <- HB.genes[!is.na(HB.genes)]

scRNA.6[["percent.HB"]]<-PercentageFeatureSet(scRNA.6, features=HB.genes)

col.num <- length(levels(scRNA.6@active.ident))

HB.genes <- c("HBA1","HBA2","HBB","HBD","HBE1","HBG1","HBG2","HBM","HBQ1","HBZ")

HB_m <- match(HB.genes, rownames(scRNA.7@assays$RNA))

HB.genes <- rownames(scRNA.7@assays$RNA)[HB_m]

HB.genes <- HB.genes[!is.na(HB.genes)]

scRNA.7[["percent.HB"]]<-PercentageFeatureSet(scRNA.7, features=HB.genes)

col.num <- length(levels(scRNA.7@active.ident))

HB.genes <- c("HBA1","HBA2","HBB","HBD","HBE1","HBG1","HBG2","HBM","HBQ1","HBZ")

HB_m <- match(HB.genes, rownames(scRNA.10@assays$RNA))

HB.genes <- rownames(scRNA.10@assays$RNA)[HB_m]

HB.genes <- HB.genes[!is.na(HB.genes)]

scRNA.10[["percent.HB"]]<-PercentageFeatureSet(scRNA.10, features=HB.genes)

col.num <- length(levels(scRNA.10@active.ident))

HB.genes <- c("HBA1","HBA2","HBB","HBD","HBE1","HBG1","HBG2","HBM","HBQ1","HBZ")

HB_m <- match(HB.genes, rownames(scRNA.11@assays$RNA))

HB.genes <- rownames(scRNA.11@assays$RNA)[HB_m]

HB.genes <- HB.genes[!is.na(HB.genes)]

scRNA.11[["percent.HB"]]<-PercentageFeatureSet(scRNA.11, features=HB.genes)

col.num <- length(levels(scRNA.11@active.ident))

HB.genes <- c("HBA1","HBA2","HBB","HBD","HBE1","HBG1","HBG2","HBM","HBQ1","HBZ")

HB_m <- match(HB.genes, rownames(scRNA.12@assays$RNA))

HB.genes <- rownames(scRNA.12@assays$RNA)[HB_m]

HB.genes <- HB.genes[!is.na(HB.genes)]

scRNA.12[["percent.HB"]]<-PercentageFeatureSet(scRNA.12, features=HB.genes)

col.num <- length(levels(scRNA.12@active.ident))

HB.genes <- c("HBA1","HBA2","HBB","HBD","HBE1","HBG1","HBG2","HBM","HBQ1","HBZ")

HB_m <- match(HB.genes, rownames(scRNA.13@assays$RNA))

HB.genes <- rownames(scRNA.13@assays$RNA)[HB_m]

HB.genes <- HB.genes[!is.na(HB.genes)]

scRNA.13[["percent.HB"]]<-PercentageFeatureSet(scRNA.13, features=HB.genes)

col.num <- length(levels(scRNA.13@active.ident))

HB.genes <- c("HBA1","HBA2","HBB","HBD","HBE1","HBG1","HBG2","HBM","HBQ1","HBZ")

HB_m <- match(HB.genes, rownames(scRNA.14@assays$RNA))

HB.genes <- rownames(scRNA.14@assays$RNA)[HB_m]

HB.genes <- HB.genes[!is.na(HB.genes)]

scRNA.14[["percent.HB"]]<-PercentageFeatureSet(scRNA.14, features=HB.genes)

col.num <- length(levels(scRNA.14@active.ident))

violin <- VlnPlot(scRNA.14,

features = c("nFeature_RNA", "nCount_RNA", "percent.mt","percent.HB"),

cols =rainbow(col.num),

pt.size = 0.01, #不需要显示点，可以设置pt.size = 0

ncol = 4) +

theme(axis.title.x=element_blank(), axis.text.x=element_blank(), axis.ticks.x=element_blank())

###把图片画到画板上面

violin

scRNA.11 <- subset(scRNA.1, subset = nFeature_RNA > 300& nFeature_RNA < 3500 & percent.mt < 10 & percent.HB < 3 & nCount_RNA < 10000)

scRNA.1

scRNA.11

scRNA.22 <- subset(scRNA.2, subset = nFeature_RNA > 300& nFeature_RNA < 3500 & percent.mt < 10 & percent.HB < 3 & nCount_RNA < 10000)

scRNA.33 <- subset(scRNA.3, subset = nFeature_RNA > 300& nFeature_RNA < 3500 & percent.mt < 10 & percent.HB < 3 & nCount_RNA < 20000)

scRNA.44 <- subset(scRNA.4, subset = nFeature_RNA > 300& nFeature_RNA < 3500 & percent.mt < 10 & percent.HB < 3 & nCount_RNA < 20000)

scRNA.55 <- subset(scRNA.5, subset = nFeature_RNA > 300& nFeature_RNA < 3500 & percent.mt < 10 & percent.HB < 3 & nCount_RNA < 20000)

scRNA.66 <- subset(scRNA.6, subset = nFeature_RNA > 300& nFeature_RNA < 3500 & percent.mt < 10 & percent.HB < 3 & nCount_RNA < 15000)

scRNA.77 <- subset(scRNA.7, subset = nFeature_RNA > 300& nFeature_RNA < 3500 & percent.mt < 10 & percent.HB < 3 & nCount_RNA < 10000)

scRNA.1010 <- subset(scRNA.10, subset = nFeature_RNA > 300& nFeature_RNA < 3500 & percent.mt < 10 & percent.HB < 3 & nCount_RNA < 10000)

scRNA.1111 <- subset(scRNA.11, subset = nFeature_RNA > 300& nFeature_RNA < 3500 & percent.mt < 10 & percent.HB < 3 & nCount_RNA < 15000)

scRNA.1212 <- subset(scRNA.12, subset = nFeature_RNA > 300& nFeature_RNA < 3500 & percent.mt < 10 & percent.HB < 3 & nCount_RNA < 10000)

scRNA.1313 <- subset(scRNA.13, subset = nFeature_RNA > 300& nFeature_RNA < 3500 & percent.mt < 10 & percent.HB < 3 & nCount_RNA < 15000)

scRNA.1414 <- subset(scRNA.14, subset = nFeature_RNA > 300& nFeature_RNA < 3500 & percent.mt < 10 & percent.HB < 3 & nCount_RNA < 10000)

scRNA_harmony <- merge(scRNA.11, y=c(scRNA.22,scRNA.33,scRNA.44,scRNA.55,scRNA.66,scRNA.77,scRNA.1010,scRNA.1111,scRNA.1212,scRNA.1313,scRNA.1414))

scRNA_harmony <- NormalizeData(scRNA_harmony) %>% FindVariableFeatures() %>% ScaleData() %>% RunPCA(verbose=FALSE)

system.time({scRNA_harmony <- RunHarmony(scRNA_harmony, group.by.vars = "orig.ident")})

###一定要指定harmony

scRNA_harmony <- FindNeighbors(scRNA_harmony, reduction = "harmony", dims = 1:25) %>% FindClusters(resolution = 1.5)

#######UMAP降维,

scRNA_harmony<- RunUMAP(scRNA_harmony, reduction = "harmony", dims = 1:25)

#######TSNE降维,运行时间久

scRNA_harmony <- RunTSNE(scRNA_harmony, reduction = "harmony", dims = 1:16)

?DimPlot#一般按照meta.data的列明进行分组画图

colnames(scRNA_harmony1@meta.data)

plot1 =DimPlot(scRNA_harmony, reduction = "umap",label = T)

plot2 = DimPlot(scRNA_harmony, reduction = "umap", group.by='orig.ident')

#combinate

plotc <- plot1+plot2

plotc

save(scRNA_harmony,file = "scRNA_harmony_UMAP.rdata")

load("scRNA_harmony_UMAP.rdata")

scRNA_harmony<-scRNA_harmony1

##高变基因是在各个维度表达值变化大，用于降维聚类，差异基因是一个维度的基因和其它维度的基因差异比较大，用的是data数据

markers <- FindAllMarkers(object = scRNA_harmony, test.use="wilcox" , ###也有ROC，MAST

only.pos = TRUE,

logfc.threshold = 0.25) ###可以修改最低0.25

all.markers =markers %>% dplyr::select(gene, everything()) %>% subset(p_val<0.05)

top10 = all.markers %>% group_by(cluster) %>% top_n(n = 10, wt = avg_log2FC)

write.table(top10,file="top10.txt")

write.csv(top10,file="top10.csv")

write.table(all.markers,file="all.markers.txt")

write.csv(all.markers,file="all.markers.csv")

###################机器注释

#第二种方法用SingleR鉴定细胞类型

BiocManager::install("SingleR")

library(SingleR)

##把师傅给你的百度云打开 下载其中的人的数据库，因为你们没有vpn，所以singler的数据库没法下载

###下载好数据库后，把ref_Human_all.Rdata加载到环境中，这样算是对数据库的加载，就可以按照singler的算法来对细胞亚群进行定义了。

load("ref_Human_all.RData")

###我们可以看到在环境中多了一个叫ref_Human_all的文件 大小为113mb 这个就是数据库

####然后我们把环境中的ref_Human_all赋值与refdata

refdata <- ref_Human_all

###把rna的转录表达数据提取

?GetAssayData

scRNA_harmony <- JoinLayers(scRNA_harmony)

testdata <- GetAssayData(scRNA_harmony, slot="data")

###把scRNA数据中的seurat_clusters提取出来，注意这里是因子类型的

clusters <- scRNA_harmony@meta.data$seurat_clusters

#######开始注释

###开始用singler分析

cellpred <- SingleR(test = testdata, ref = refdata, labels = refdata$label.main,

method = "cluster", clusters = clusters,

assay.type.test = "logcounts", assay.type.ref = "logcounts")

plotScoreHeatmap(cellpred )########Singer注释评价指标

###制作细胞类型的注释文件

celltype = data.frame(ClusterID=rownames(cellpred), celltype=cellpred$labels, stringsAsFactors = FALSE)

###保存一下

write.csv(celltype,"celltype_singleR.csv",row.names = FALSE)

##把singler的注释写到metadata中 有两种方法

###方法一

scRNA_harmony@meta.data$celltype = "NA"

for(i in 1:nrow(celltype)){

scRNA_harmony@meta.data[which(scRNA_harmony@meta.data$seurat_clusters == celltype$ClusterID[i]),'celltype'] <- celltype$celltype[i]}

###因为我把singler的注释加载到metadata中时候，命名的名字叫celltype，所以画图时候，group.by="celltype"

DimPlot(scRNA_harmony, group.by="celltype", label=T, label.size=5)

###方法二：

celltype = data.frame(ClusterID=rownames(cellpred), celltype=cellpred$labels, stringsAsFactors = F)

scRNA_harmony@meta.data$singleR=celltype[match(clusters,celltype$ClusterID),'celltype']

###因为我把singler的注释加载到metadata中时候，命名的名字叫singleR，所以画图时候，group.by="singleR"

DimPlot(scRNA_harmony, group.by="singleR", label=T, label.size=5)

###我们可以看到 两种方法得到的结果都是一样的，但是我比较喜欢第二种方法

##################重新写入新定义的细胞类型#############

celltype<-read.csv('celltype_singleR1.csv',header = T)

scRNA_harmony@meta.data$celltype = "NA"

for(i in 1:nrow(celltype)){

scRNA_harmony@meta.data[which(scRNA_harmony@meta.data$seurat_clusters == celltype$ClusterID[i]),'celltype'] <- celltype$celltype[i]}

###因为我把singler的注释加载到metadata中时候，命名的名字叫celltype，所以画图时候，group.by="celltype"

DimPlot(scRNA_harmony, group.by="celltype", label=T, label.size=5)

###保存注释后的文件

save(scRNA_harmony,file = "scRNA_harmony_ZHUSHI.rdata")

load("scRNA_harmony_ZHUSHI.rdata")

table(scRNA_harmony$orig.ident)

Idents(scRNA_harmony)="celltype"

prop.table(table(Idents(scRNA_harmony)))

table(Idents(scRNA_harmony), scRNA_harmony$orig.ident)

Cellratio <- prop.table(table(Idents(scRNA_harmony), scRNA_harmony$orig.ident), margin = 2)

Cellratio <- as.data.frame(Cellratio)

colourCount = length(unique(Cellratio$Var1))

ggplot(Cellratio) +

geom_bar(aes(x =Var2, y= Freq, fill = Var1),stat = "identity",width = 0.7,size = 0.5,colour = '#222222')+

theme_classic() +

labs(x='Sample',y = 'Ratio')+

coord_flip()+

theme(panel.border = element_rect(fill=NA,color="black", size=0.5, linetype="solid"))

allcolour=c("#DC143C","#0000FF","#20B2AA","#FFA500","#9370DB","#98FB98","#F08080","#1E90FF","#7CFC00","#FFFF00",

"#808000","#FF00FF","#FA8072","#7B68EE","#9400D3","#800080","#A0522D","#D2B48C","#D2691E","#87CEEB","#40E0D0","#5F9EA0",

"#FF1493","#0000CD","#008B8B","#FFE4B5","#8A2BE2","#228B22","#E9967A","#4682B4","#32CD32","#F0E68C","#FFFFE0","#EE82EE",

"#FF6347","#6A5ACD","#9932CC","#8B008B","#8B4513","#DEB887")

ggplot(Cellratio) +

geom_bar(aes(x =Var2, y= Freq, fill = Var1),stat = "identity",width = 0.7,size = 0.5,colour = '#222222')+

theme_classic() +

labs(x='Sample',y = 'Ratio')+

scale_fill_manual(values = allcolour)+

theme(panel.border = element_rect(fill=NA,color="black", size=0.5, linetype="solid"),

axis.text.x = element_text(angle = 45, hjust = 1),

axis.title.x = element_text(face = "bold"), # 设置x轴标题为加粗

axis.title.y = element_text(face = "bold")) # 设置y轴标题为加粗)

write.csv(Cellratio,'细胞比例数据.csv',row.names = TRUE)

B "CD79A","CD79B" EN "CLDN5", "VWF", "PECAM1" EP "CAPS", "SNTN" 巨噬细胞 "CD80","CD86","CD163"

单核 "CD14" TT "CD3D" gan "ALDH1A1","PTPRC"

marker <- c("CD79A","CD79B","CLDN5", "VWF", "PECAM1","CAPS", "SNTN","CD80","CD86","CD163","CD14","CD3D","ALDH1A1","PTPRC")

DotPlot(scRNA_harmony, features = marker)+coord_flip()+

theme_bw()+

theme(panel.grid = element_blank(), axis.text.x=element_text(hjust = 1,vjust=0.5))+

labs(x=NULL,y=NULL)+guides(size=guide_legend(order=3))+

scale_color_gradientn(values = seq(0,1,0.2),colours = c('#330066','#336699','#66CC66','#FFCC33'))

FeaturePlot(scRNA_harmony, features = c("IL1RAP"), max.cutoff = 3,cols = c("grey", "red"))

select_genes1 <- c('IL1RAP')

DotPlot(scRNA_harmony, features = select_genes1,assay = 'RNA',cols = c('blue', 'red'))+RotatedAxis()

#############################自定义画图

#对clus命名

Idents(scRNA_harmony)= "celltype"

##改为样本名字

Idents(scRNA1)= "orig.ident"

##Dim画图默认的分组信息是Idents。

DimPlot(scRNA_harmony)

degs <- FindAllMarkers(object = scRNA_harmony, test.use="wilcox" , ###也有ROC，MAST

only.pos = TRUE,

logfc.threshold = 0.25) ###可以修改最低0.25

all.markers =degs %>% dplyr::select(gene, everything()) %>% subset(p_val<0.05)

top10 = all.markers %>% group_by(cluster) %>% top_n(n = 10, wt = avg_log2FC)

write.table(top10,file="top10.txt")

write.csv(top10,file="top10.csv")

write.table(all.markers,file="all.markers.txt")

write.csv(all.markers,file="all.markers.csv")

#############################原版适配#######################

####找Marker基因

degs <- FindAllMarkers(scRNA_harmony, logfc.threshold = 0.5,

test.use = "roc",

return.thresh = 0.25,

min.pct = 0.3, only.pos = T)

##挑选出Top基因

degs_sig <- degs %>%

filter(pct.1 > 0.3 &

power > 0.25) %>%

filter(cluster != "other") %>%

arrange(cluster, -power)

# select degs for heatmap

degs_top50 <- degs_sig %>%

# filter(cluster!="other") %>%

group_by(cluster) %>%

top_n(50, power) %>%

top_n(50, avg_diff) %>%

arrange(cluster, -power)

###去平均值

avgData <- scRNA_harmony@assays$RNA@data[degs_top50$gene,] %>%

apply(1, function(x){

tapply(x, scRNA_harmony$celltype, mean) # ExpMean

}) %>% t

##对挑选出的基因scale，同时限制基因的表达最大和最小值

phData <- MinMax(scale(avgData), -2, 2) # z-score

rownames(phData) <- 1:nrow(phData)

######画热图

##install.packages("pheatmap")

library(pheatmap)

phres <- pheatmap(

phData,

color = colorRampPalette(c("darkblue", "white", "red3"))(99), #配色

scale = "row",

cluster_rows = F, #不按行聚类

cluster_cols = T, #按列聚类

clustering_method = "complete",

show_rownames = F, #显示cluster名

annotation_row = data.frame(cluster = degs_top50$cluster),

)

###优化，按照大小重新排序

phData1= phData[, c("Epithelial_cells","T_cells","Endothelial_cells","Macrophage","Tissue_stem_cells","B_cell","Monocyte","CMP")]

phres <- pheatmap(

phData1,

color = colorRampPalette(c("darkblue", "white", "red3"))(99), #配色

scale = "row",

cluster_rows = F, #不按行聚类

cluster_cols = F, #按列聚类

clustering_method = "complete",

show_rownames = F, #显示cluster名

annotation_row = data.frame(cluster = degs_top50$cluster),

)
