# Supplementary figures and images for: Integrated single-cell and transcriptome sequencing data reveal the value of IL1RAP in gastric cancer microenvironment and prognosis
Source: Front Oncol. 2025 May 15;15:1584619. doi: 10.3389/fonc.2025.1584619 (PMC12119286; doi:10.3389/fonc.2025.1584619)

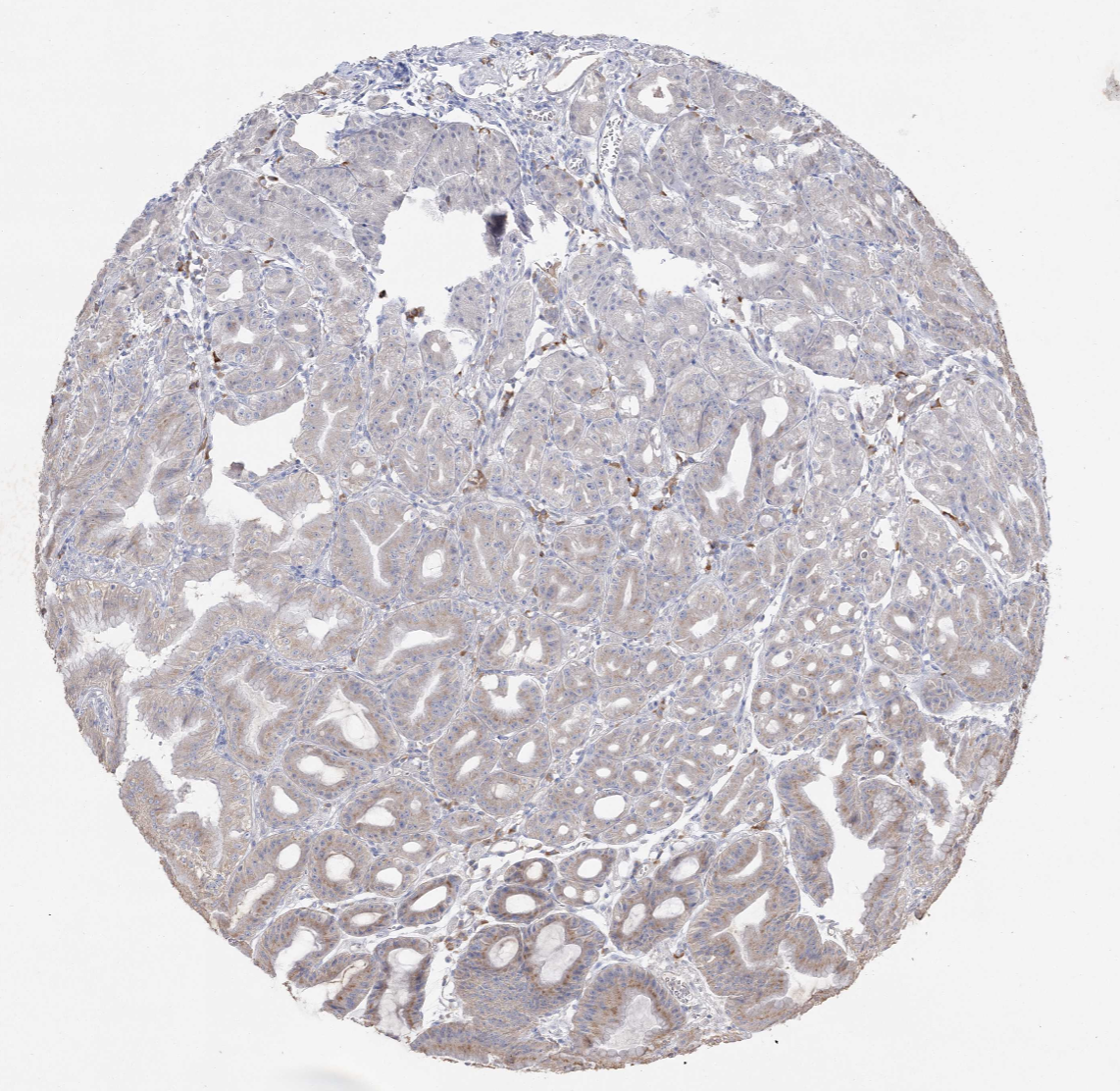

Supplement: Supplementary file 10 [file Image1.png]

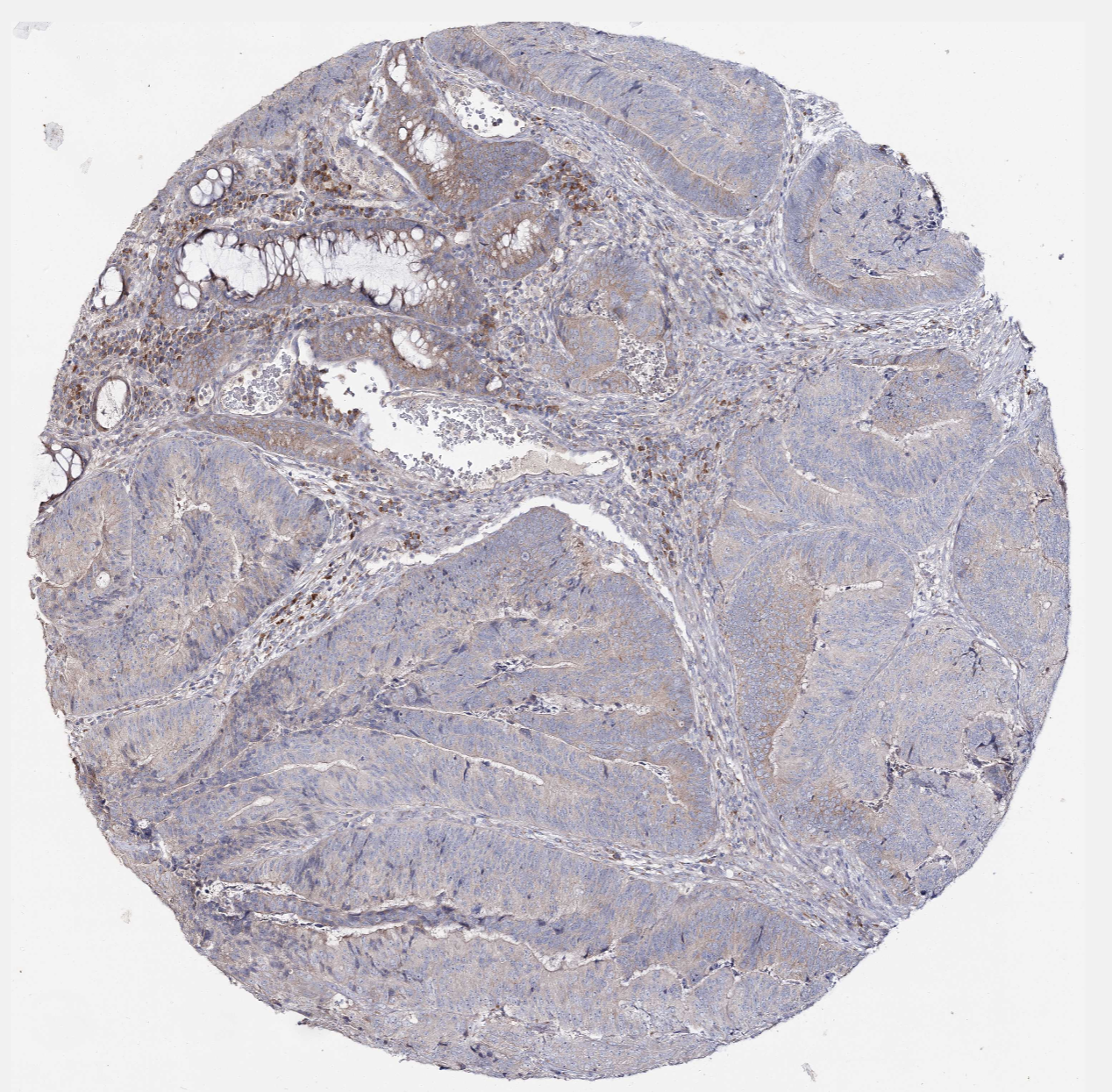

Supplement: Supplementary file 11 [file Image2.png]
